# Obstetricians’ recognition and attitudes towards guidelines for managing group B Streptococcus-positive pregnant women in Japanese maternity homes: A nationwide study

**DOI:** 10.18332/ejm/212553

**Published:** 2025-10-31

**Authors:** Kotomi Yamaguchi, Kazutomo Ohashi

**Affiliations:** 1 Graduate School of Nursing, Nagoya City University, Nagoya, Japan; 2 Otemae University, Osaka, Japan

**Keywords:** group B Streptococcus, midwives, obstetricians, guidelines, Japan

## Abstract

**INTRODUCTION:**

Midwives working at Japanese maternity homes are permitted to manage deliveries of women with group B Streptococcus (GBS), provided there is collaboration with obstetricians, as outlined in the Japanese Midwives Association (JMA) guidelines. However, obstetricians’ recognition of the guidelines remains limited, potentially affecting the continuity and safety of perinatal care.

**METHODS:**

A nationwide cross-sectional survey was conducted in 2017. Anonymous self-administered questionnaires were mailed to all 2423 obstetric institutions accredited by the Japan Council for Quality Health Care, and responses from obstetricians were analyzed. Analyses used SPSS v23.0; p<0.05 was considered significant. The survey assessed obstetricians’ recognition of the JMA guidelines, experience with midwife-led deliveries, and attitudes towards neonatal GBS prevention.

**RESULTS:**

Valid responses from 941 obstetricians from the 2423 institutions (38.8%) were analyzed. Only 31.9% (300/941) of the obstetricians reported being aware of the JMA guidelines, and just 15.1% (142/941) correctly understood that midwives may manage GBS-positive deliveries. Obstetricians with experience as a commissioned doctor for maternity homes demonstrated significantly higher awareness and fewer concerns regarding midwife-led care (chi-squared test, p<0.05). Discrepancies in transfer decisions and neonatal management were observed, particularly when maternal fever or prolonged membrane rupture occurred.

**CONCLUSIONS:**

Although the relatively low response rate may limit the generalizability of the findings, we determined that obstetricians’ limited recognition of the JMA guidelines may hinder effective interprofessional collaboration in maternity homes. Promoting mutual understanding through interprofessional education and establishing standardized clinical protocols for GBS-related care are essential to improve continuity and safety in maternal and neonatal care in Japan.

## INTRODUCTION

Early-onset group B Streptococcus (EOGBS) infection is a significant concern in perinatal care, particularly regarding preventive strategies and interprofessional collaboration. In 2002, the Centers for Disease Control and Prevention (CDC) published guidelines recommending universal GBS screening and intrapartum antibiotic prophylaxis (IAP) for pregnant women^1^. Consequently, the incidence of EOGBS markedly declined from 1.7 to 0.34–0.37 per 1000 births^2^. Since then, the combination of screening and IAP has remained the only consistently effective strategy for EOGBS prevention^3,4^.

The Japan Society of Obstetrics and Gynecology (JSOG) established EOGBS prevention guidelines, introduced nationwide, based on the CDC policy in 2008^5^, and in 2014, the Japanese Midwives Association (JMA) revised its midwifery practice guidelines, stating that when GBS-positive women deliver at a maternity home, collaboration with an obstetrician and pediatrician and compliance with the JSOG guidelines are mandatory^6^. As midwives are the main care providers for low-risk deliveries in maternity homes, the JMA guidelines are important to ensure maternal and neonatal safety and to promote collaboration between professionals. Although the JMA guidelines have not been revised since 2014, subsequent international and domestic updates have been made. In the United States, the American Academy of Pediatrics and the American College of Obstetricians and Gynecologists jointly issued revised guidelines in 2019, introducing a standardized neonatal management algorithm^7^. The JSOG updated its guidelines by changing the recommended screening period from 33 to 35 weeks of gestation^8^. Perspectives of pediatricians on neonatal management of GBS-positive deliveries have been investigated in a separate nationwide survey, which has already been published^9^. Subsequent nationwide surveys showed no significant change in the incidence of EOGBS (0.12/1000 live births in 2004–2008 vs 0.09/1000 in 2009–2010), but neonatal mortality decreased from 14.8% to 11.8%^10^.

Surveys of midwives in 2015^11^ and pediatricians in 2016^9^ highlighted persistent challenges in guideline implementation. In a 2015 survey of midwives in which 66.2% handled deliveries of GBS-positive women, only 47.5% adhered to the screening timing recommended in the guidelines and 58.5% had prior consultation with the commissioned obstetrician^11^. In addition, in a 2016 survey of pediatricians, 26.4% were aware of the existence of the JMA guidelines and only 9.8% knew that midwives at maternity homes could handle deliveries of GBS-positive women^9^. Such discrepancies in professional awareness and practice may also compromise the continuity and safety of care for both mothers and neonates towards the prevention and early detection of EOGBS.

Midwife-led delivery care may offer numerous benefits, including higher maternal satisfaction, improved birth outcomes, and reduced medical interventions^12,13^. Notably, Nove et al.^14^ suggested that expanding access to skilled midwifery care could substantially reduce maternal and neonatal mortality, as well as stillbirths. In Japan, where low-risk births at maternity homes are legally permissible under certain conditions, ensuring safe GBS-positive deliveries requires robust interprofessional collaboration and a shared understanding of guidelines between midwives, obstetricians, and pediatricians. Particularly, to support continuity and safety in neonatal care, enhanced mutual understanding of roles and improved information-sharing among different professional groups are required.

In the context of managing deliveries of GBS-positive women in maternity homes, the extent to which obstetricians recognize the JMA guidelines, respond appropriately to GBS-positive women and their newborns, and understand EOGBS detection remains unclear. Furthermore, the 2017 revision of the JSOG guidelines changed the recommended timing for GBS screening from after 33 weeks to after 35 weeks of gestation^8^, potentially increasing the difficulty of scheduling the test appropriately in clinical practice. In the US, the revised 2019 American College of Obstetricians and Gynecologists guidelines further narrowed the screening window from 36 weeks 0 days to 37 weeks 6 days, placing greater emphasis on the validity of the results at the time of labor^7^. In contrast, GBS prevention strategies in Europe vary considerably: countries such as France and Germany follow universal screening policies, while the United Kingdom continues to adopt a risk-based approach^15^. Recent reports in Japan indicate that the incidence of neonatal GBS infection has remained largely unchanged, underscoring the urgent need to improve preventive strategies and strengthen interprofessional coordination^16^.

Therefore, based on the results of a 2017 nationwide survey, we aim to clarify obstetricians’ awareness of the JMA guidelines, their approaches to managing GBS-positive mothers and their newborns at maternity homes, and their perceptions of EOGBS.

## METHODS

This study was conducted and reported in accordance with the Strengthening the Reporting of Observational Studies in Epidemiology guidelines.

### Design and setting

This was a cross-sectional survey. An anonymous, self-administered questionnaire and explanatory document were mailed to the obstetric departments at 2423 facilities registered with the Japan Council for Quality Health Care (covering approximately 85.7% of all deliveries in Japan), between April and May 2017. Responses were obtained from representative obstetricians at each institution.

### Participants

All obstetricians working at facilities that handled deliveries were eligible. Exclusion criteria included obstetricians who reported not handling deliveries and questionnaires returned with blank responses.

### Variables and measurements

The questionnaire covered respondent characteristics (years of practice as an obstetrician, experience with neonatal GBS disease treatment, treatment of neonates transferred from maternity homes, experience of introduction to a pediatrician in such situations, and experience as a commissioned obstetrician for maternity homes), institutional characteristics (annual live birth numbers, number of obstetricians), and awareness and understanding of the JMA guidelines. Outcomes of interest included: 1) recognition of the existence of the JMA guidelines; 2) understanding of guideline content; 3) opinions on transfer decisions outlined in the JMA guidelines; and 4) perspectives on routine tests for neonates born to GBS-positive women, with or without IAP (Table 1). A ‘commissioned obstetrician’ is an obstetrician formally designated by a maternity home to provide medical support and assume responsibility in the event of complications during delivery.

**Table 1:** Overview of recommendations for the management of pregnant women with group B Streptococcus infection in Japan, extracted from the guidelines of the Japan Society of Obstetrics and Gynecology and the Japanese Midwives Association

*Guidelines*	*Key Points*
**JSOG guidelines**	1. GBS screening is performed at 33–37 weeks of gestation (this guideline was revised to ‘between 35 and 37 weeks of gestation’ in 2017). 2. Collect a specimen for GBS culture via a lower vaginal and anal canal swab. 3. Administer prophylactic antibiotic treatment to GBS-positive women who are in labor and scheduled to give birth via vaginal delivery or after premature rupture of membranes during pregnancy.
**JMA guidelines**	Midwives can manage the delivery of GBS-positive pregnant women at maternity homes without obstetrician supervision only if they comply with the JSOG guidelines and cooperate with commissioned obstetricians to prepare for unforeseeable accidents. The JMA guidelines were issued in 2014 and had remained unchanged as of 2024. In the following two cases, GBS-positive pregnant women in labor are transported to a hospital by ambulance: 1. Passing of ≥18 hours since membrane rupture. 2. Maternal body temperature of ≥38.0℃.

GBS: group B Streptococcus. JSOG: Japan Society of Obstetrics and Gynecology. JMA: Japanese Midwives Association. Adapted from the Guidelines for Obstetric and Gynecologic Practice^5,8^ and the Guidelines for Midwives Practice^6^. Information is presented as of 2024.

An original questionnaire was designed to assess routine perspectives on the necessary tests for neonates born to GBS-positive women, with or without IAP, based on previous studies.

### Bias

As this was a self-administered survey, potential biases include non-response bias, self-reporting bias, and recall bias. To minimize these risks, questionnaires were anonymized and distributed to all eligible facilities nationwide.

### Sample size

All 2423 accredited facilities were invited to participate. As this was a nationwide survey of all eligible institutions, no prior sample size calculation was performed.

### Statistical analysis

Data were summarized using descriptive statistics. After excluding missing values, analysis was performed using SPSS Statistics 23.0 software (IBM Japan Ltd., Tokyo, Japan). A chi-squared test was used to examine the relationship between knowledge or attitude and experience as a commissioned doctor. The significance level was set at 5.0%.

### Ethical considerations

This study was approved by the Ethics Committee of Kyoto University Medical School (approval number: R0946). Informed consent forms were mailed, and consent was implied upon questionnaire return. Anonymity was maintained throughout. This study adhered to the principles of the Declaration of Helsinki.

## RESULTS

### Sample characteristics

Nine hundred and forty-six obstetricians from the 2423 facilities (39.0%) responded to the survey. After excluding three who did not handle deliveries and two whose questionnaires had blank responses, 941 were included in the analysis (valid response rate: 38.8%). Obstetricians’ mean duration of practice was 28.0 ± 10.0 years (n=922). The total number of deliveries, average annual deliveries, and number of obstetricians at each facility were: 388016; 421.1 ± 308.3 (range: 5–3400) (n=915); and 4.3 ± 4.8 (range: 1–60) (n=922), respectively. Of the obstetricians, 9.0% (85/941) were currently commissioned doctors, 3.9% (37/941) had past experience, and 85.0% (800/941) had never held this position; 2.0% (19/941) did not respond or gave other answers. Furthermore, 33.2% (312/941) had experience in treating neonates with GBS, and 11.8% (111/941) had treated neonates transported from maternity homes with abnormal conditions. Obstetricians who referred neonates to pediatricians, regardless of the GBS diagnosis, accounted for 94.9% (296/312 obstetricians with experience in treating neonates with GBS). Furthermore, 83.8% (93/111 obstetricians who had treated neonates transported from maternity homes) of the obstetricians referred neonates diagnosed with GBS to pediatricians.

### JSOG guidelines

Of the obstetricians included in the analysis, 96.3% (906/941) agreed with universal GBS screening, while 2.3% (22/941) considered it unnecessary.

### JMA guidelines

Three hundred obstetricians out of 941 (31.9%) recognized the existence of the JMA guidelines. Additionally, 15.1% (142/941) were familiar with the content of the JMA guidelines, which stipulate that midwives at maternity homes are responsible for managing the labor of GBS-positive women.

### Relationship between experience and recognition of JMA guidelines

Among obstetricians with experience as commissioned doctors, 58.8% (70/119) were aware of the existence of the JMA guidelines. In contrast, 28.8% (219/542) of those without such experience reported awareness (p<0.001). With regard to the revised content of the JMA guidelines, 40.0% (48/120) of those with experience as commissioned doctors understood the revised content of the JMA guidelines, compared with 11.7% (89/760) of those without such experience (p<0.001). When asked about concerns regarding midwifery management at maternity homes, 65.8% (449/682) of obstetricians without experience as commissioned doctors were more inclined to consider that there were ‘certain general problems regarding the management of midwives at maternity homes’ than those with experience (54.8%; 57/104) (p=0.036) (Table 2).

**Table 2:** Comparison of recognition of the Japanese Midwives Association guidelines according to experience as a commissioned doctor

*Items*	*Response*	*All n (%)*	*Commissioned or ex-commissioned experience n (%)*	*No commissioned obstetrician experience n (%)*	p*
Are you aware of the existence of the JMA guidelines?	Yes	289 (32.8)	70 (58.8)	219 (28.8)	<0.001
	No	591 (67.2)	49 (41.2)	542 (71.2)	
	Total	880 (100)	119 (13.5)	761 (86.5)	
Do you know whether midwives at maternity homes deal with the labor of GBS-positive women in accordance with JMA guidelines?	Yes	137 (15.6)	48 (40.0)	89 (11.7)	<0.001
	No	743 (84.4)	72 (60.0)	671 (88.3)	
	Total	880 (100)	120 (13.6)	760 (86.4)	
There are some problems in midwives’ management of labor at maternity homes.	Yes	506 (64.4)	57 (54.8)	449 (65.8)	0.036
	No	280 (35.6)	47 (45.2)	233 (34.2)	
	Total	786 (100)	104 (13.2)	682 (86.8)	

JMA: Japanese Midwives Association.

* χ² test was used. This information is based on a nationwide cross-sectional survey of obstetricians at obstetric delivery facilities accredited by the Japan Council for Quality Health Care (n=941), conducted in April–May 2017.

### Opinions about routine management of neonates born to GBS-positive women

**Figure 1:**
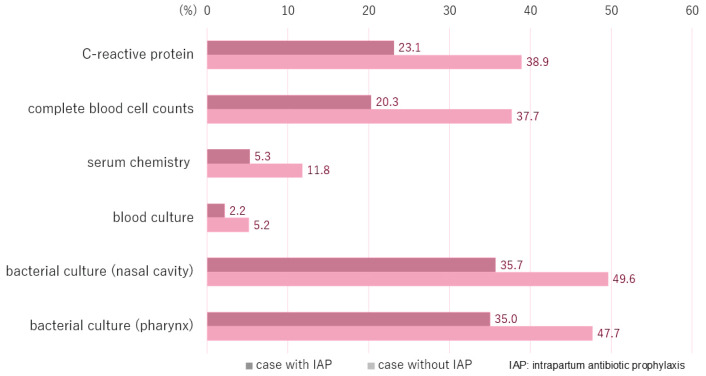
Proportion of obstetricians (N=941: multiple responses permitted) who considered each routine clinical test necessary for neonates born to group B Streptococcus-positive women, with and without intrapartum antibiotic prophylaxis, based on a nationwide cross-sectional survey of representative obstetricians at obstetric delivery facilities accredited by the Japan Council for Quality Health Care (April-May 2017).

Of the 941 obstetricians, 709 (75.3%) considered the guidelines necessary for the management of neonates born to GBS-positive women. Subsequently, which routine clinical tests obstetricians deemed essential for neonates born to GBS-positive women, both with and without IAP administration, were determined. When IAP was administered, the three tests most frequently selected as necessary routine clinical tests were nasal cavity cultures (35.7%; 336/941), serum C-reactive protein (CRP) level analysis (23.1%; 217/941), and complete blood count analysis (20.3%; 191/941). Conversely, when IAP was not administered, 49.6% (467/941) of the obstetricians deemed nasal cavity culture testing necessary, 38.9% (366/941) chose CRP, and 37.7% (355/941) recommended complete blood count analysis (Figure 1).

### Transfer

We asked obstetricians about their judgment regarding the transfer of a GBS-positive pregnant woman in labor from a maternity home to a hospital as outlined in the JMA guidelines.

In cases where more than 18 hours had passed since membrane rupture, 46.7% (439/941) believed that the pregnant woman should be transported to a hospital, while 35.8% (337/941) believed they should consult a commissioned doctor or pediatrician from a commissioned medical institution. In instances of maternal body temperature exceeding 38.0°C, 76.0% (715/941) considered transferring the mother to a hospital, and 14.9% (140/941) chose consulting a commissioned doctor or pediatrician from a commissioned medical institution. The full distribution of responses for each condition is presented in Table 3.

**Table 3:** Obstetricians’ opinions regarding the judgment of transfer of group B Streptococcuspositive women in delivery from maternity homes to hospitals

*Management*	*n (%)*
**Ambulance transport in case of more than 18 hours having passed since rupture of the membranes**	
Should be transported to the hospital	439 (46.7)
Consult commissioned obstetricians and pediatricians at cooperating medical facilities	337 (35.8)
Don’t know	70 (7.4)
Other	63 (6.7)
No answer	32 (3.4)
**Ambulance transport in case of maternal body temperature more than 38.0°C**	
Should be transported to the hospital	715 (76.0)
Consult commissioned obstetricians and pediatricians at cooperating medical facilities	140 (14.9)
Don’t know	20 (2.1)
Other	33 (3.5)
No answer	33 (3.5)

GBS: group B Streptococcus. This information is based on a nationwide cross-sectional survey of obstetricians at obstetric delivery facilities accredited by the Japan Council for Quality Health Care (n=941), conducted in April–May 2017.

## DISCUSSION

In this study, we explored obstetricians’ awareness and understanding of the JMA guidelines regarding the delivery management of GBS-positive women at maternity homes. We also examined their approaches to neonatal care and perceptions of EOGBS infection. The findings highlight current challenges and future directions for enhancing safe, midwife-led maternity care and improving neonatal outcomes through interprofessional collaboration.

### Obstetricians’ recognition and understanding of the JMA guidelines

Of the obstetricians, only 31.9% were aware of the existence of the JMA guidelines, and just 15.1% accurately understood their content. This limited awareness may reflect the midwife-specific focus of the guidelines, relatively low prevalence of midwife-led births, and insufficient interprofessional communication in daily practice.

In Japan, maternity homes are required to appoint commissioned obstetricians to support midwives in managing delivery-related complications. When more complex cases exceeding the capacity of the commissioned doctor arise, a hospital with pediatric services must be designated^17^. Although this framework is intended to facilitate collaboration, its success often depends on individual relationships, and continuous communication systems are not always in place. A previous study reported that only 58.3% of maternity homes had held discussions with their commissioned obstetricians about managing GBS-positive women^11^, indicating gaps in practical coordination despite formal arrangements. In the present study, obstetricians with experience as commissioned doctors demonstrated significantly greater awareness and understanding of the JMA guidelines than did those without such experience. This suggests that actual clinical collaboration between obstetricians and midwives plays a key role in improving interprofessional understanding of midwife-led protocols. In addition, we consider that regular meetings between midwives and commissioned obstetricians could provide opportunities for case-based discussion and help improve shared awareness of the guidelines, while interprofessional dialogue is essential for consistent guideline implementation.

A previous study also revealed variation among pediatricians in the timing of neonatal assessment for infants born to GBS-positive mothers^9^. The close connection between intrapartum management and neonatal care underscores the importance of information-sharing across professions.

Gaps in interprofessional communication may impede consistent adherence to guidelines. Previous studies have identified barriers such as limited awareness, weak communication, and a lack of institutional support^16,18^. Interventions such as academic detailing have been shown to significantly improve GBS screening compliance^19^, highlighting the need for ongoing interprofessional education and structured knowledge exchange.

Lack of awareness is not confined to obstetricians; low levels of guideline recognition among pediatricians have been reported^9^. As both professions are essential to the care of neonates born to GBS-positive mothers, the lack of a shared understanding points to inadequate interprofessional communication regarding implementation of GBS-related care.

Effective collaboration between physicians and nursing professionals is critical for preventing and managing EOGBS. There is a need for structured frameworks that foster mutual understanding and timely information-sharing across all healthcare professionals involved in GBS care. These findings align with those of a Cochrane review showing that interprofessional collaboration improves clinical practice and outcomes^20^.

### Midwife-led care for GBS-positive women: benefits and challenges

Midwifery-led models of care offer numerous benefits for both mothers and infants, such as increased maternal satisfaction and reduced rates of medical intervention^12,13^. In light of the JMA guideline revision, the development of a care system for GBS-positive women that centers on collaboration between midwives and other healthcare professionals is expected to enhance the quality and continuity of neonatal care.

Despite the existence of supportive policies and evidence, we found that awareness of the JMA guidelines remains limited among obstetricians, and many continue to express concern or hesitation about midwife-led delivery care for GBS-positive women. Among obstetricians without prior experience as commissioned doctors, 65.8% indicated concerns about midwifery management at maternity homes, compared with 54.8% of those with such experience. This suggests that the level of concern may relate more to the degree of interprofessional collaboration experience than to midwife-led care itself.

Effective implementation of midwife-led care requires not only institutional structures but also mutual understanding and acceptance from collaborating obstetricians, as emphasized by recent international recommendations^21^. The World Health Organization’s ‘Global Strategic Directions for Nursing and Midwifery 2021–2025’ emphasizes the need to strengthen interprofessional collaboration and education to ensure safe and coordinated care. A 2021 review also reported that simulation-based training and team-based learning improved collaborative practice and patient outcomes in obstetric care^22^. Together, these approaches – including structured meetings and simulation-based education – represent feasible strategies to improve interprofessional awareness. In light of these international developments, the present study, together with previous nationwide survey data, can serve as a useful foundation for considering institutional frameworks and the introduction of interprofessional education in obstetric and neonatal care in Japan. The present findings indicate that obstetricians with experience working alongside midwives are less likely to express concerns, underscoring the importance of fostering interprofessional understanding through practical collaboration. Midwives should therefore take an active role in sharing the content and scientific rationale of the JMA guidelines with obstetricians. However, awareness of the JMA guidelines is disproportionately limited to professionals working within maternity home settings, and may not be widely disseminated among other healthcare providers. Based on our results, we suggest that it is necessary to establish systems for interprofessional information-sharing so that GBS-related guidelines can be consistently applied across all care settings.

### Transfer decisions and guideline compliance for GBS-positive mothers and their newborns

Among obstetricians, 83.8% reported referring abnormal neonates transferred from maternity homes to pediatricians, and 94.9% did so when the newborns were diagnosed with GBS. These findings suggest a moderate level of collaboration between obstetricians and pediatricians in the initial management of high-risk neonates. However, there was notable variability in adherence to the JMA’s transport criteria: 76.0% of obstetricians supported transfer when maternal temperature reached ≥38.0°C, while only 46.7% did so when ≥18 hours had passed since membrane rupture. A similar pattern was observed in a previous national survey of midwives^8^, highlighting a potential gap in interpreting and applying the criteria.

Such inconsistencies may arise from the nature of the criteria themselves. While maternal fever is a straightforward clinical indicator, the ‘≥18 hours since rupture of membranes’ threshold requires more nuanced documentation and greater clinical judgment. Internationally, difficulties in applying transport protocols have been linked to discrepancies between guideline content and the realities of clinical decision-making^23^. Abstract or rigid transport criteria may also contribute to inconsistent decision-making across professionals, leading to potential delays or safety risks.

To improve consistency in transfer decisions, the criteria must be revisited and practice-oriented protocols that account for variability in clinical judgment must be developed. Simulation-based training^22^ using case scenarios and regular opportunities for interprofessional discussion of GBS-related guidelines may support shared understanding and improve maternal and neonatal outcomes.

### Perspectives of obstetricians and pediatricians on neonatal secondary GBS prevention

Regarding the secondary prevention of EOGBS infection in newborns, whether those born to GBS-positive mothers or healthy newborns whose mothers received inadequate IAP, the CDC does not recommend routine testing, citing a lack of sufficient clinical evidence to support its effectiveness^2^. As of 2025, no standardized clinical algorithm for the postnatal management of GBS-exposed newborns has been established in Japan. In recent international developments, the American Academy of Pediatrics and American College of Obstetricians and Gynecologists introduced a newborn management algorithm in their 2019 guidelines^14,24^. This algorithm is based on risk stratification using factors such as maternal GBS colonization status, presence or absence of IAP, duration since membrane rupture, and maternal body temperature. However, no standardized neonatal algorithm currently exists in Japan. In 2017, the JSOG revised its guideline by shifting the recommended screening period from 33 to 35 weeks of gestation, but the JMA guideline has not been updated since 2014. As a result, clinical management is often left to the discretion of individual healthcare providers, increasing the risk of variation in practice and potentially leading to both under- and overtreatment of newborns. This underscores the continued importance of the present findings as baseline data to inform future revisions of domestic guidelines. Blood cultures can help avoid unnecessary antibiotic use. However, from a clinical standpoint, the biomarkers currently in use lack sufficient reliability to ensure a definitive diagnosis^25,26^.

This study revealed notable differences in clinical judgment between obstetricians and pediatricians regarding the appropriate testing for newborns born to GBS-positive mothers. Pediatricians tended to prioritize blood-based diagnostics such as blood cultures and CRP tests, whereas obstetricians emphasized bacterial cultures from the upper respiratory tract more^9^. These discrepancies likely reflect the differing clinical frameworks and risk assessment approaches between specialties. However, they also underscore a broader systemic issue: the absence of standardized neonatal care protocols for newborns exposed to GBS in Japan. This lack of standardization significantly burdens midwives and nursing staff, who are responsible for initial postnatal observation and early intervention when abnormalities occur.

Indeed, both this study and a previous national survey^9^ showed that the majority of obstetricians and pediatricians recognize the need for clear, unified guidelines for the preventive management of these newborns. In this context, the present study, in which we examined the actual conditions of care from a multidisciplinary perspective, can serve as valuable foundational data for developing standardized protocols and improving clinical practices.

The establishment of nationwide and practice-oriented guidelines for the secondary prevention of EOGBS is an urgent priority. Particularly, to enable nurses and midwives to make appropriate clinical decisions and ensure maternal and neonatal safety in daily practice, it is essential to strengthen collaboration with obstetricians and pediatricians, develop actionable guidelines based on shared understanding, and implement a sustainable system for ongoing interprofessional dialogue and education. In Europe, postnatal management strategies for GBS-exposed neonates vary across countries owing to the absence of a unified guideline, highlighting the importance of context-specific approaches^27^. This variation indicates that countries must tailor GBS prevention strategies to their unique healthcare settings and challenges.

This study is the only nationwide survey in Japan to include midwives, pediatricians, and obstetricians, and the focus on obstetricians’ recognition of and attitudes towards the JMA guidelines can continue to provide useful data for evaluating and revising collaborative care systems. The findings offer practical insights into interprofessional collaboration and may inform efforts to improve neonatal GBS prevention. The differences observed between professional groups may stem from variations in role expectations and interprofessional engagement, indicating the need for more consistent opportunities for collaboration and education to support uniform implementation of guidelines.

## Limitations

This study has several limitations. First, the response rate was relatively low (38.8%); therefore, the generalizability of the findings is limited. In addition, selection bias may have occurred if institutions or physicians with a particular interest in GBS management were more likely to respond, and the possibility of recall bias cannot be excluded because responses relied on self-reporting. Furthermore, incomplete or missing data in some questionnaires may have constrained the analysis. However, the annual number of births at the responding facilities represented approximately 40.0% of all deliveries nationwide, suggesting that the results retain a certain level of validity regarding facility coverage and delivery volume.

Second, this survey was conducted in 2017 and may not fully reflect the current clinical landscape. Nevertheless, the JMA guidelines have not been revised since 2014, and implementation challenges remain. Moreover, there are still very few studies^28,29^ – either in Japan or internationally – that have explored the management of GBS-positive women and their newborns from a multi-professional perspective. Therefore, despite the time gap, these findings remain valuable as the only nationwide data available on obstetricians’ perspectives, providing important baseline information for evaluating future changes. In this context, the present study offers rare and useful findings into interprofessional collaboration in perinatal care.

### Implications for practice and further research

Although the revised JMA guidelines emphasize the importance of multidisciplinary collaboration in the delivery care of GBS-positive women, this study revealed variations in understanding and awareness among obstetricians. Given that multiple healthcare professionals are involved in the care of GBS-positive mothers and their newborns, it is essential to establish a system for continuous information-sharing and to promote a shared understanding of the guidelines among all relevant professionals.

Furthermore, in the absence of national guidelines for the secondary prevention of EOGBS, clinical judgement and practices tend to vary among professional groups, making it difficult to ensure consistency in neonatal care. To safeguard the health of both mothers and newborns, it is necessary to create regular forums for multidisciplinary dialogue aimed at aligning clinical practices and standardizing evidence-based care. Further studies are warranted to explore how interprofessional understanding and collaboration can be effectively strengthened across diverse clinical settings in the care of GBS-positive mothers and their newborns.

## CONCLUSIONS

This study revealed that obstetricians have limited awareness of the JMA guidelines concerning the management of labor in GBS-positive women. Variations among obstetricians in their understanding of appropriate care for newborns born to GBS-positive women were also identified. Considered together with findings from previous surveys of midwives and pediatricians, the results suggest interprofessional differences in clinical judgment and responses related to the care of GBS-positive women and their newborns. Such discrepancies may affect the timely detection of EOGBS and consistent implementation of secondary prevention strategies.

To ensure safe and consistent neonatal care, it is essential to disseminate GBS-related guidelines to all healthcare professionals involved in perinatal and neonatal care, strengthen interprofessional collaboration, and develop unified and practice-based care protocols.

## Data Availability

The data supporting this research cannot be made available for privacy or other reasons.
